# Supplementary material for: Viral RNA load in plasma is associated with critical illness and a dysregulated host response in COVID-19
Source: Crit Care. 2020 Dec 14;24:691. doi: 10.1186/s13054-020-03398-0 (PMC7734467; doi:10.1186/s13054-020-03398-0)
Supplement: Supplementary file 1 — Additional file 1. Recruited patients in each hospital. [file 13054_2020_3398_MOESM1_ESM.docx]

| Hospital | Outpatients | Wards | ICU |
| --- | --- | --- | --- |
| Hospital Clínico Universitario de Valladolid | 0 | 0 | 45 |
| Hospital General Universitario Gregorio Marañón (Madrid) | 44 | 4 | 25 |
| Hospital Universitario Infanta Leonor (Madrid) | 0 | 30 | 0 |
| Hospital Universitario y Politécnico de La Fe (Valencia) | 0 | 27 | 0 |
| Hospital Universitario Rio Hortega (Valladolid) | 2 | 22 | 23 |
| Hospital Universitario Príncipe de Asturias (Madrid) | 4 | 17 | 1 |
| Hospital Universitario de Burgos | 0 | 0 | 2 |
| Hospital Universitario de León | 0 | 0 | 4 |
| Total | 50 | 100 | 100 |

**Additional file 1: recruited patients in each hospital**
