# Supplementary material for: Viral RNA load in plasma is associated with critical illness and a dysregulated host response in COVID-19
Source: Crit Care. 2020 Dec 14;24:691. doi: 10.1186/s13054-020-03398-0 (PMC7734467; doi:10.1186/s13054-020-03398-0)
Supplement: Supplementary file 2 — Additional file 2. Multivariate logistic regression analysis comparing outpatients against wards patients (Enter method). The association between viral RNAemia with hospitalization at the wards was evaluated adjusting by major confounding factors. [file 13054_2020_3398_MOESM2_ESM.docx]

|  | **OR [CI 95%]** | ***p*** |
| --- | --- | --- |
| Age (years) | 1.071 [1.024 - 1.121] | 0.003 |
| Obesity | 5.618 [0.841 - 37.552] | 0.075 |
| Hypertension | 1.024 [0.279 - 3.756] | 0.972 |
| Dyslipidemia | 1.521 [0.354 - 6.532] | 0.573 |
| O2 saturation | 1.077 [0.946 - 1.226] | 0.261 |
| Bilateral infiltrate | 7.652 [2.050 - 28.558] | 0.002 |
| Glucose (mg/dl) | 1.022 [0.992 - 1.054] | 0.156 |
| INR | 3.346 [0.121 - 92.829] | 0.476 |
| D Dimer (pg/ml) | 1.000 [1.000 - 1.000] | 0.111 |
| LDH (UI/L) | 1.005 [0.998 - 1.012] | 0.176 |
| CRP (mg/dl) | 1.032 [1.012 - 1.052] | 0.002 |
| Lymphocytes (cells/mm3) | 1.000 [0.998 - 1.001] | 0.440 |
| Monocytes (cells/mm3) | 0.998 [0.996 - 1.001] | 0.223 |
| Neutrophils (cells/mm3) | 1.000 [1.000 - 1.000] | 0.382 |
| Viral RNAemia | 3.793 [0.344 - 41.845] | 0.277 |

**Additional file 2. Multivariate logistic regression analysis comparing outpatients against wards patients (Enter method).** The association between viral RNAemia with hospitalization at the wards was evaluated adjusting by major confounding factors.
