# Supplementary material for: Viral RNA load in plasma is associated with critical illness and a dysregulated host response in COVID-19
Source: Crit Care. 2020 Dec 14;24:691. doi: 10.1186/s13054-020-03398-0 (PMC7734467; doi:10.1186/s13054-020-03398-0)
Supplement: Supplementary file 3 — Additional file 3. Multivariate logistic regression analysis comparing outpatients against wards patients (backward stepwise selection method/Likelihood Ratio). The association between viral RNAemia with hospitalization at the wards was evaluated adjusting by major confounding factors, but it was not selected in the final model. [file 13054_2020_3398_MOESM3_ESM.docx]

|  | **OR [CI95%]** | ***p*** |
| --- | --- | --- |
| Age | 1.057 [1.017 - 1.098] | 0.005 |
| Obesity | 6.675 [1.129 - 39.472] | 0.036 |
| Bilateral infiltrate | 6.400 [2.153 - 19.024] | 0.001 |
| Glucose (mg/dl) | 1.027 [0.999 - 1.055] | 0.058 |
| CRP (mg/dl) | 1.032 [1.013 -1.051] | 0.001 |
| Monocytes (cells/mm3) | 0.997 [0.995 - 1.000] | 0.017 |

**Additional file 3. Multivariate logistic regression analysis comparing outpatients against wards patients (backward stepwise selection method / Likelihood Ratio).** The association between viral RNAemia with hospitalization at the wards was evaluated adjusting by major confounding factors, but it was not selected in the final model.
