# Supplementary material for: Viral RNA load in plasma is associated with critical illness and a dysregulated host response in COVID-19
Source: Crit Care. 2020 Dec 14;24:691. doi: 10.1186/s13054-020-03398-0 (PMC7734467; doi:10.1186/s13054-020-03398-0)
Supplement: Supplementary file 4 — Additional file 4. Multivariate logistic regression analysis comparing wards patients against ICU patients (backward stepwise selection method/Likelihood Ratio). The association between viral RNAemia and viral RNA load with critical illness was evaluated adjusting by major confounding factors. [file 13054_2020_3398_MOESM4_ESM.docx]

|  | **OR [CI95%]** | | | **p** | **OR [CI95%]** | | | **p** | **OR [CI95%]** | | | **p** |
| --- | --- | --- | --- | --- | --- | --- | --- | --- | --- | --- | --- | --- |
| Systolic Pressure (mmHg) | 0.976 | 0.953 | 1.000 | 0.049 | 0.974 | 0.949 | 0.999 | 0.040 | 0.974 | 0.950 | 0.999 | 0.042 |
| Glucose (mg/dl) | 1.006 | 1.000 | 1.012 | 0.050 | 1.006 | 1.000 | 1.012 | 0.055 | 1.005 | 0.999 | 1.012 | 0.088 |
| D-dimer (pg/mL) | 1.000 | 1.000 | 1.000 | 0.057 | 1.000 | 1.000 | 1.000 | 0.025 | 1.000 | 1.000 | 1.000 | 0.088 |
| LDH (UI/L) | 1.008 | 1.004 | 1.011 | 0.000 | 1.006 | 1.003 | 1.010 | 0.001 | 1.007 | 1.004 | 1.011 | 0.000 |
| Haematocrit (%) | 0.798 | 0.717 | 0.888 | 0.000 | 0.789 | 0.706 | 0.881 | 0.000 | 0.775 | 0.688 | 0.873 | 0.000 |
| Lymphocytes (cells/mm3) | 0.997 | 0.996 | 0.999 | 0.000 | 0.997 | 0.996 | 0.999 | 0.000 | 0.997 | 0.996 | 0.999 | 0.000 |
| **Viral RNAemia (Yes)** | 4.270 | 1.575 | 11.580 | 0.004 |  |  |  |  |  |  |  |  |
| **Viral RNA load (N1)**  **in plasma, log (copies/mL)** |  |  |  |  | 2.005 | 1.350 | 2.978 | 0.001 |  |  |  |  |
| **Viral RNA load (N2)**  **in plasma, log (copies/mL)** |  |  |  |  |  |  |  |  | 2.071 | 1.419 | 3.022 | 0.000 |

**Additional file 4. Multivariate logistic regression analysis comparing wards patients against ICU patients (backward stepwise selection method / Likelihood Ratio).** The association between viral RNAemia and viral RNA load with critical illness was evaluated adjusting by major confounding factors.
