# Supplementary material for: Viral RNA load in plasma is associated with critical illness and a dysregulated host response in COVID-19
Source: Crit Care. 2020 Dec 14;24:691. doi: 10.1186/s13054-020-03398-0 (PMC7734467; doi:10.1186/s13054-020-03398-0)
Supplement: Supplementary file 5 — Additional file 5. Laboratory parameters levels across groups. [file 13054_2020_3398_MOESM5_ESM.docx]

|  | **Healthy Controls**  **(0)** | **Outpatients**  **(1)** | **Ward**  **(2)** | **ICU**  **(3)** | ***p***  **(0 vs 1)** | ***p***  **(0 vs 2)** | ***p***  **(0 vs 3)** | ***p***  **(1 vs 2)** | ***p***  **(1 vs 3)** | ***p***  **(2 vs 3)** |
| --- | --- | --- | --- | --- | --- | --- | --- | --- | --- | --- |
| **ICAM-1**  **(pg/ml)** | 289208 [92857] | 358431.50 [88702.30] | 377655 [150081.80] | 466818 [202189.80] | 0.036 | < 0.001 | < 0.001 | n.s. | < 0.001 | < 0.001 |
| **Lipocalin-2**  **(pg/ml)** | 82634.50  [29205.30] | 73134.50  [32007.30] | 94077  [49868] | 100165  [57819.80] | n.s. | n.s. | n.s. | 0.011 | < 0.001 | n.s. |
| **Myeloperoxidase**  **(pg/ml)** | 27541.50  [12906.80] | 41902  [30413.80] | 88244.50  [78145] | 141971.50  [148623.50] | n.s. | < 0.001 | < 0.001 | < 0.001 | < 0.001 | < 0.001 |
| **VCAM-1**  **(pg/ml)** | 618380  [195510.50] | 800393  [403378] | 1191387  [618827] | 1248699.50  [603679] | 0.021 | < 0.001 | < 0.001 | < 0.001 | < 0.001 | n.s. |
| **PD-L1**  **(pg/ml)** | 55.85  [33.27] | 110.50  [120.68] | 178  [114.25] | 228  [137.75] | 0.031 | < 0.001 | < 0.001 | 0.045 | < 0.001 | 0.031 |
| **G-CSF**  **(pg/ml)** | 6.90  [7.67] | 23.55  [20.48] | 31.95  [42.85] | 36.25  [53.35] | < 0.001 | < 0.001 | < 0.001 | n.s. | 0.022 | n.s. |
| **IL-1b**  **(pg/ml)** | 0.44  [1.65] | 0.16  [0.30] | 0.44  [0.53] | 0.38  [0.51] | n.s. | n.s. | n.s. | 0.005 | < 0.001 | n.s. |
| **IL-10**  **(pg/ml)** | 1.59  [0.48] | 4.19  [6.68] | 9.11  [10.14] | 17  [22.25] | 0.002 | < 0.001 | < 0.001 | 0.007 | < 0.001 | < 0.001 |
| **IL-17A**  **(pg/ml)** | 1.95  [1.08] | 2.38  [1.45] | 2.32  [1.44] | 3.06  [2.87] | n.s. | 0.047 | 0.002 | n.s. | n.s. | n.s. |
| **GM-CSF**  **(pg/ml)** | 0.66  [0.42] | 1.07  [0.54] | 1.10  [0.67] | 1.62  [1.01] | n.s. | n.s. | < 0.001 | n.s. | < 0.001 | < 0.001 |
| **IL-7**  **(pg/ml)** | 1.81  [1.49] | 2.04  [3.12] | 3.19  [4.19] | 4.84  [5.17] | n.s. | 0.009 | < 0.001 | 0.017 | < 0.001 | n.s. |
| **CXCL10**  **(pg/ml)** | 91.9  [71.85] | 470.50  [731.50] | 977.50  [943] | 1609.50  [1693.75] | 0.003 | < 0.001 | < 0.001 | 0.002 | < 0.001 | 0.003 |
| **Angiopoietin-2**  **(pg/ml)** | 708  [260.75] | 809  [479.75] | 1128  [779.50] | 1769.50  [2064.25] | n.s. | 0.001 | < 0.001 | 0.008 | < 0.001 | 0.001 |
| **IL-1ra**  **(pg/ml)** | 478  [1159.75] | 653.50  [1039.25] | 1796  [1896] | 1634  [4472.50] | n.s. | < 0.001 | < 0.001 | < 0.001 | < 0.001 | n.s |
| **IL-6**  **(pg/ml)** | 1.27  [0.83] | 6.34  [12.42] | 33.25  [48.10] | 105.50  [252.23] | 0.027 | < 0.001 | < 0.001 | < 0.001 | < 0.001 | < 0.001 |
| **CCL2**  **(pg/ml)** | 103.50  [39] | 189  [157.50] | 283.50  [304] | 490  [881.75] | 0.012 | < 0.001 | < 0.001 | 0.031 | < 0.001 | < 0.001 |
| **IL-12p70**  **(pg/ml)** | 0.62  [0.71] | 1.46  [0.67] | 1.51  [0.71] | 1.36  [0.94] | < 0.001 | < 0.001 | < 0.001 | n.s. | n.s. | n.s. |
| **IL-2**  **(pg/ml)** | 0.02  [0.09] | 0.53  [0.39] | 0.49  [0.57] | 0.63  [0.83] | < 0.001 | < 0.001 | < 0.001 | n.s. | n.s. | n.s. |
| **IL-4**  **(pg/ml)** | 0.13  [0.27] | 0.33  [0.34] | 0.33  [0.39] | 0.29  [0.37] | 0.021 | 0.006 | 0.041 | n.s. | n.s. | n.s. |
| **IL-15**  **(pg/ml)** | 2.11  [0.54] | 3.85  [3.7] | 4.89  [2.90] | 8.38  [5.19] | 0.001 | < 0.001 | < 0.001 | n.s. | < 0.001 | < 0.001 |
| **Granzyme-B**  **(pg/ml)** | 12  [15.75] | 33.20  [27.92] | 46.15  [40.03] | 39.25  [35.58] | < 0.001 | < 0.001 | < 0.001 | n.s. | n.s. | 0.036 |
| **INFγ**  **(pg/ml)** | 0.68  [0.45] | 4.35  [14.16] | 7.42  [15.13] | 3.44  [8.50] | < 0.001 | < 0.001 | < 0.001 | n.s. | n.s. | 0.021 |
| **TNFα**  **(pg/ml)** | 5.11  [1.79] | 10.60  [4.18] | 13  [6.46] | 12.95  [10.34] | < 0.001 | < 0.001 | < 0.001 | 0.021 | 0.006 | n.s. |

**Additional file 5. Laboratory parameters ‘levels across groups.**
